# Development of preoperative and postoperative models to predict recurrence in postoperative glioma patients: a longitudinal cohort study

**DOI:** 10.1186/s12885-024-11996-2

**Published:** 2024-02-28

**Authors:** Wanyu Qiao, Yi Wang, Chen Luo, Jinsong Wu, Guoyou Qin, Jie Zhang, Ye Yao

**Affiliations:** 1 Department of Biostatistics, School of Public Health & National Clinical Research Center for Aging and Medicine, Huashan Hospital, Fudan University, Shanghai, China; 2 Department of Tumor Screening and Prevention, Zhongshan Hospital, Fudan University, Shanghai, China; 3 Department of Neurosurgery, Huashan Hospital, Shanghai Medical College, Fudan University, Shanghai, China; 4 Neurosurgical Institute, Fudan University, Shanghai, China; 5 Shanghai Clinical Medical Center of Neurosurgery, Shanghai, China; 6 Shanghai Key Laboratory of Brain Function and Restoration and Neural Regeneration, Shanghai, China; 7 National Clinical Research Centre for Aging and Medicine, Huashan Hospital, Fudan University, Shanghai, China; 8 Key Laboratory of Public Health Safety of Ministry of Education, Fudan University, Shanghai, China

**Keywords:** Postoperative glioma recurrence, Predictive model, Risk factors, Cox regression

## Abstract

**Background:**

Glioma recurrence, subsequent to maximal safe resection, remains a pivotal challenge. This study aimed to identify key clinical predictors influencing recurrence and develop predictive models to enhance neurological diagnostics and therapeutic strategies.

**Methods:**

This longitudinal cohort study with a substantial sample size (*n* = 2825) included patients with non-recurrent glioma who were pathologically diagnosed and had undergone initial surgical resection between 2010 and 2018. Logistic regression models and stratified Cox proportional hazards models were established with the top 15 clinical variables significantly influencing outcomes screened by the least absolute shrinkage and selection operator (LASSO) method. Preoperative and postoperative models predicting short-term (within 6 months) postoperative recurrence in glioma patients were developed to explore the risk factors associated with short- and long-term recurrence in glioma patients.

**Results:**

Preoperative and postoperative logistic models predicting short-term recurrence had accuracies of 0.78 and 0.87, respectively. A range of biological and early symptomatic characteristics linked to short- and long-term recurrence have been pinpointed. Age, headache, muscle weakness, tumor location and Karnofsky score represented significant odd ratios (t > 2.65, *p* < 0.01) in the preoperative model, while age, WHO grade 4 and chemotherapy or radiotherapy treatments (t > 4.12, *p* < 0.0001) were most significant in the postoperative period. Postoperative predictive models specifically targeting the glioblastoma and IDH wildtype subgroups were also performed, with an AUC of 0.76 and 0.80, respectively. The 50 combinations of distinct risk factors accommodate diverse recurrence risks among glioma patients, and the nomograms visualizes the results for clinical practice. A stratified Cox model identified many prognostic factors for long-term recurrence, thereby facilitating the enhanced formulation of perioperative care plans for patients, and glioblastoma patients displayed a median progression-free survival (PFS) of only 11 months.

**Conclusion:**

The constructed preoperative and postoperative models reliably predicted short-term postoperative glioma recurrence in a substantial patient cohort. The combinations risk factors and nomograms enhance the operability of personalized therapeutic strategies and care regimens. Particular emphasis should be placed on patients with recurrence within six months post-surgery, and the corresponding treatment strategies require comprehensive clinical investigation.

**Supplementary Information:**

The online version contains supplementary material available at 10.1186/s12885-024-11996-2.

## Background

Gliomas, the most prevalent and aggressive primary central nervous system (CNS) tumors [1], represent about 75% of malignant primary brain tumors in adults [2], with poor prognosis and high recurrence rate [3]. In China, the annual incidence rate of glioma is reported to be 5–8 per 100,000 individuals, with a five-year mortality rate ranking third among systemic tumors, surpassed only by pancreatic and lung cancer [4].

A combination therapy approach involving maximal safe surgical resection, adjuvant radiotherapy, and chemotherapy with temozolomide has shown promise in extending patient survival [5–7]. However, tumor recurrence remains a significant challenge, with some patients experiencing relapse within six months after surgery [8]. The time interval between initial surgery and recurrence has important prognostic value for patients, and consideration of subsequent treatment options for patients at different risk of recurrence is also highly prioritized [9, 10]. For those patients with a high likelihood of recurrence in the short term, the value of surgery, radiotherapy or conservative treatment needs to be individualized and explored. Identifying short-term recurrence and the patient’s varying risk of recurrence is therefore important and may affect the final medical management and treatment plan, as well as the patient’s physical, psychological, and emotional trajectory, and even quality of life. Models based on vital clinical variables that predict glioma recurrence may provide favorable support in medical practice management consulting services. Other investigations [11, 12] propose that the deployment of machine learning or deep learning algorithms in radiomics could enhance model performance. However, these approaches necessitate a considerable number of standardized, high-quality images and intricate algorithmic refinements, often implicating a degree of overfitting, thereby circumscribing their broad clinical application.

The patient’s biological and early clinical signs (e.g., muscle weakness, seizures), which may be indicative of tumor recurrence [13–15], denote an adjusted plan of care and further investigations. Factors associated with tumor recurrence in patients with glioma include age, sex, the degree of tumor resection, tumor location, and isocitrate dehydrogenase (IDH) status [16–18]. Different combinations of these characteristics are the main causes of varying prognoses, and exploring different combinations of risk factors will inform the physician’s personalized treatment plan and postoperative risk management plan for the patient. However, most studies have only discerned a plethora of individual risk factors, without taking into consideration the influence of frequently encountered combinations of diverse risk factors on prognosis. Previous studies probing risk factors for glioma recurrence often featured limited sample sizes, concentrated predominantly on glioblastoma, and evaluated risk factors predictive of overall survival rather than progression-free survival (PFS).

To this end, the current study aims to discern critical, readily accessible clinical variables and integrate them into models predictive of postoperative recurrence or mortality in glioma patients. Patients with recurrent gliomas typically present a dismal prognosis, with limited effective therapeutic options. Given this scenario, patient preferences for specific treatment strategies warrant extensive exploration, with the objective of suggesting novel avenues for subsequent clinical decision-making post-glioma recurrence. This endeavor could potentially be realized through a preoperative prediction model, particularly for patients at high risk of recurrence or mortality within six months post-surgery. The findings of this investigation could conceivably offer guidance for therapeutic trajectories concerning patients of a challenging nature, allowing for the incorporation of select statistical facets to substantiate clinical deliberations.

## Methods

### Study design and participants

Patients with a pathological diagnosis of glioma who underwent tumor resection at Huashan Hospital, Fudan University between March 2010 and December 2018 were screened for this study (as per WHO CNS tumor classification, version 2016). Of the total study population of 2563 patients who met the inclusion criteria (eFigure 1 in the appendix), 167 were either lost to follow-up or declined further participation within six months post-surgery without definitive recurrence or mortality data; these patients were excluded from the Logistic model (Dataset 1), but were included in the Cox model (Dataset 2) as right-censored data. The primary outcome, progression-free survival (PFS), was delineated as the interval from tumor surgical resection to the date of glioma recurrence or death from any cause, with recurrence verified according to the Response Assessment in Neuro-Oncology (RANO) criteria [19]. Consequently, the primary endpoints for Dataset 1 and Dataset 2 were identified as 6-month PFS and long-term PFS, respectively. The study protocol received approval from the Huashan Hospital ethics review committee. Written informed consent was obtained from all participants.

### Data collection and variable selection

Univariate analyses of 58 variables revealed significant differences between postoperative recurrence and control groups for the majority of variables (Table 1). To construct predictive models incorporating critical clinical parameters, the Least Absolute Shrinkage and Selection Operator (LASSO) method [20] was deployed to sequence variables and filter the top 15, inclusive of sex and age at diagnosis. Logistic models and corresponding nomograms were formulated to predict postoperative recurrence within 6 months and elucidate potential risk factors. Stratified Cox proportional hazards models and correlating nomograms were employed to assess the influence of multiple variables on the time to glioma recurrence or mortality.

**Table 1:** Demographic and clinical data of glioma patients with postoperative recurrence and controls

Demographic and Clinical Data	PR^a^ (*n* = 367)	Controls(*n* = 2029)	t/Z/χ^2^	*p* value
**Preoperative Period**				
Age at Diagnosis (Years)	54.61 (13.29), 6.00–78.00	43.25 (13.93), 3.00–82.00	t = 14.95	< 0.0001^****^
Sex male No. (%)	245 (67)	1152 (57)	χ^2^ = 12.74	3.59 × 10^− 4***^
Illness Duration (Months)	3.97 (15.74), 0.10–240.00	5.73 (14.40), 0.05–240.00	t = -1.96	0.05
Headache No. (%)	184 (50)	754 (37)	χ^2^ = 21.96	< 0.0001^****^
Dizziness No. (%)	44 (12)	288 (14)	χ^2^ = 1.27	0.26
Nausea No. (%)	16 (4)	75 (4)	χ^2^ = 0.37	0.54
Vomiting No. (%)	32 (9)	142 (7)	χ^2^ = 1.37	0.24
Epilepsy No. (%)	8 (2)	161 (8)	χ^2^ = 15.70	< 0.0001^****^
Intracranial Space-Occupying Lesion or Intracranial Tumor^a^ No. (%)	6 (2)	125 (6)	χ^2^ = 12.32	4.49 × 10^− 4***^
Impaired Consciousness No. (%)	41 (11)	416 (21)	χ^2^ = 17.53	< 0.0001^****^
Muscle Twitching No. (%)	39 (11)	439 (22)	χ^2^ = 23.59	< 0.0001^****^
Muscle Weakness No. (%)	84 (23)	232 (11)	χ^2^ = 35.61	< 0.0001^****^
Limb Numbness No. (%)	23 (6)	125 (6)	χ^2^ = 0.01	0.94
Speech Disorder No. (%)	46 (13)	145 (7)	χ^2^ = 12.30	4.54 × 10^− 4***^
Memory Deterioration No. (%)	32 (9)	78 (4)	χ^2^ = 16.86	< 0.0001^****^
Slow Reaction No. (%)	22 (6)	31 (2)	χ^2^ = 28.67	< 0.0001^****^
Visual Impairment No. (%)	20 (5)	90 (4)	χ^2^ = 0.73	0.39
Lethargy No. (%)	4 (1)	9 (0)	Z = -1.54	0.12
Family History of Glioma No. (%)	1 (0)	0 (0)	Z = -1.42	0.15
Respiratory Diseases No. (%)	0 (0)	0 (0)	--	--
Digestive Diseases No. (%)	1 (0)	0 (0)	Z = -1.45	0.15
Urinary System Diseases No. (%)	0 (0)	2 (0)	Z = 0.00	1.00
Hematological Disorders No. (%)	0 (0)	0 (0)	--	--
Endocrine Diseases No. (%)	0 (0)	1 (0)	Z = 0.00	1.00
Cardiovascular Diseases No. (%)	1 (0)	3 (0)	Z = -0.71	0.47
Previous Surgical History No. (%)	13 (4)	21 (1)	χ^2^ = 13.75	2.09 × 10^− 4***^
History of Head Trauma No. (%)	0 (0)	6 (0)	Z = -0.53	0.60
Tumor Location				
Frontal lobe No. (%)	155 (42)	1112 (55)	χ^2^ = 19.71	< 0.0001^****^
Parietal lobe No. (%)	55 (15)	274 (14)	χ^2^ = 0.58	0.45
Occipital lobe No. (%)	38 (10)	122 (6)	χ^2^ = 9.40	2.17 × 10^− 3**^
Temporal lobe No. (%)	152 (41)	580 (29)	χ^2^ = 24.12	< 0.0001^****^
Insular lobe No. (%)	23 (6)	161 (8)	χ^2^ = 1.22	0.27
Corpus Callosum No. (%)	30 (8)	64 (3)	χ^2^ = 20.78	< 0.0001^****^
Thalamus No. (%)	15 (4)	31 (2)	χ^2^ = 10.81	1.01 × 10^− 3**^
Basal ganglia No. (%)	6 (2)	37 (2)	χ^2^ = 0.06	0.80
Brainstem No. (%)	0 (0)	10 (0)	Z = -0.88	0.38
Cerebellum No. (%)	6 (2)	47 (2)	χ^2^ = 0.67	0.41
Ventricle No. (%)	12 (3)	61 (3)	χ^2^ = 0.07	0.79
Karnofsky Score^ab^ (%)	80.98 (14.88), 30.00-100.00	84.91 (12.03), 40.00-100.00	t = 4.77	< 0.0001^****^
Postoperative Period				
WHO Grade 1/2/3/4	1/24/42/300	73/896/403/657	χ^2^ = 327.14	< 0.0001^****^
WHO Grade 1 No. (%)	1 (0)	73 (4)	χ^2^ = 11.48	7.03 × 10^− 4***^
WHO Grade 2 No. (%)	24 (7)	896 (55)	χ^2^ = 185.95	< 0.0001^****^
WHO Grade 3 No. (%)	42 (11)	403 (20)	χ^2^ = 14.56	1.36 × 10^− 4***^
WHO Grade 4 No. (%)	300 (82)	657 (32)	χ^2^ = 315.70	< 0.0001^****^
GFAP + ^a^ No. (%)	345 (96)	1948 (97)	χ^2^ = 1.47	0.23
OLIG2 + ^a^ No. (%)	244 (68)	1593 (80)	χ^2^ = 25.16	< 0.0001^****^
P53 + ^a^ No. (%)	197 (55)	923 (47)	χ^2^ = 9.20	2.43 × 10^− 3**^
IDH-1/IDH-2 + ^a^ No. (%)	34 (12)	798 (49)	χ^2^ = 129.91	< 0.0001^****^
Ki-67^a^ ≥ 20% No. (%)	99 (28)	294 (15)	χ^2^ = 36.78	< 0.0001^****^
TERT + ^a^ No. (%)	79 (54)	483 (54)	χ^2^ = 0.01	0.91
1p/19q Codeletion^a^ No. (%)	2 (2)	92 (12)	χ^2^ = 12.16	4.87 × 10^− 4***^
MGMT + ^a^ No. (%)	95 (33)	638 (42)	χ^2^ = 8.27	4.03 × 10^− 3**^
EGFR + ^a^ No. (%)	28 (93)	84 (73)	χ^2^ = 5.57	0.02^*^
Hospitalization Days	19.58 (10.16), 3.00–99.00	18.05 (8.07), 2.00–131.00	t = -2.70	0.01^*^
Chemotherapy/Radiotherapy No. (%)	237 (65)	1674 (83)	χ^2^ = 61.86	< 0.0001^****^
Karnofsky Score^ab^ (%)	82.35 (18.93), 30.00-100.00	90.63 (12.87), 20.00-100.00	t = 7.52	< 0.0001^****^
ECOG Grade^a^	1.19 (0.91), 0.00–4.00	0.76 (0.70), 0.00–4.00	t = -7.92	< 0.0001^****^

Continuous data are shown as mean (SD), minimum and maximum values in patients with postoperative recurrence and controls with statistical significance based on two sample t test. Categorical data differences (No. and percentages) in patients with postoperative recurrence and controls are represented with statistical significance based on chi-squared test (χ^2^ & p) and Fisher exact test (Z & p). *: *p* < 0.05, **: *p* < 0.01, ***: *p* < 0.001, ****: *p* < 0.0001

a: The majority of intracranial space-occupying lesion or intracranial tumor were discovered incidentally through accidental injury seeking medical attention or physical examination. PR, postoperative recurrence or death; GFAP+, glial fibrillary acidic protein positive; OLIG2+, oligodendrocyte transcription factor 2 overexpression; P53+, tumor suppressor gene p53 mutation; IDH-1/IDH-2+, isocitrate dehydrogenase 1 or isocitrate dehydrogenase 2 mutation; Ki-67, cell proliferation antigen Ki-67; TERT+, telomerase reverse transcriptase promoter mutation; 1p/19q codeletion, complete deletion of complete deletion of both the short arm of chromosome 1 (1p) and the long arm of chromosome 19 (19q); MGMT+, O-6-methylguanine-DNA methyltransferase promoter methylation; EGFR+, epidermal growth factor receptor mutation. Karnofsky score refers to an eleven levels rating scale which ranges from normal functioning (100%) to dead (0%) in 10% increments. ECOG Grade refers to a scoring system that measures the extent to which cancer affects a patient’s daily living abilities (performance status) on a scale ranging from 0 (fully active) to 5 (dead).b: 366 patients with PR and 2011 controls attended preoperative KPS score tests, while 319 patients with PR and 1911 controls participated in the postoperative KPS score tests

### Statistical analysis

Continuous data is displayed as mean ± standard deviation (SD), as well as minima and maxima, and was compared by Student’s t-tests. Categorical data in patients with postoperative recurrence and controls was examined by Chi-square tests and Fisher exact tests, as appropriate. Clinical variables were screened and prioritized using the LASSO method. ROC curves were employed to evaluate the sensitivity and specificity of the prediction models with permutation tests, and the DeLong’s test was implemented in supplementary materials to compare the AUCs (area under the ROC curves) of two different models. Significant differences and effect sizes of feature combinations were analyzed by Chi-square tests and Cohen’s w [21]. Stratified Cox regression models were deployed to satisfy the proportional hazard hypothesis determined by the Schoenfeld residuals test [22]. The Kaplan-Meier PFS curves of each group were plotted correspondingly, and the log-rank test was used to evaluate the survival differences between the groups. Statistical significance was denoted by *p* < 0.05. Statistical analyses were performed using SAS 9.4 and survival package in R version 4.0.5 software.

## Results

### Characteristics of patients

A total of 2,396 glioma patients were included in the study, comprising 1,397 (58.31%) men and 999 (41.69%) women, with a mean age of 44.99 ± 14.42 years. Of these patients, 367 (15.32%) reached the study endpoint of recurrence or death within six months after surgery, including 317 (86.38%) who relapsed and 165 (44.96%) who died. The remaining 2,029 (84.68%) patients neither relapsed nor died within the six-month postoperative period. Both the postoperative recurrence and control groups were predominantly male (245/367 [66.76%] vs. 1,152/2,029 [56.78%], χ^2^ = 12.74, *p* < 0.001). The postoperative recurrence group had a higher mean age compared to the control group (mean [SD] age, 54.61 [13.29] vs. 43.25 [13.93] years, t = 14.95, *p* < 0.001). Detailed characteristics of the patients and corresponding statistical results from univariate analyses are summarized in Table 1.

### Logistic regression analysis

**Fig. 1:**
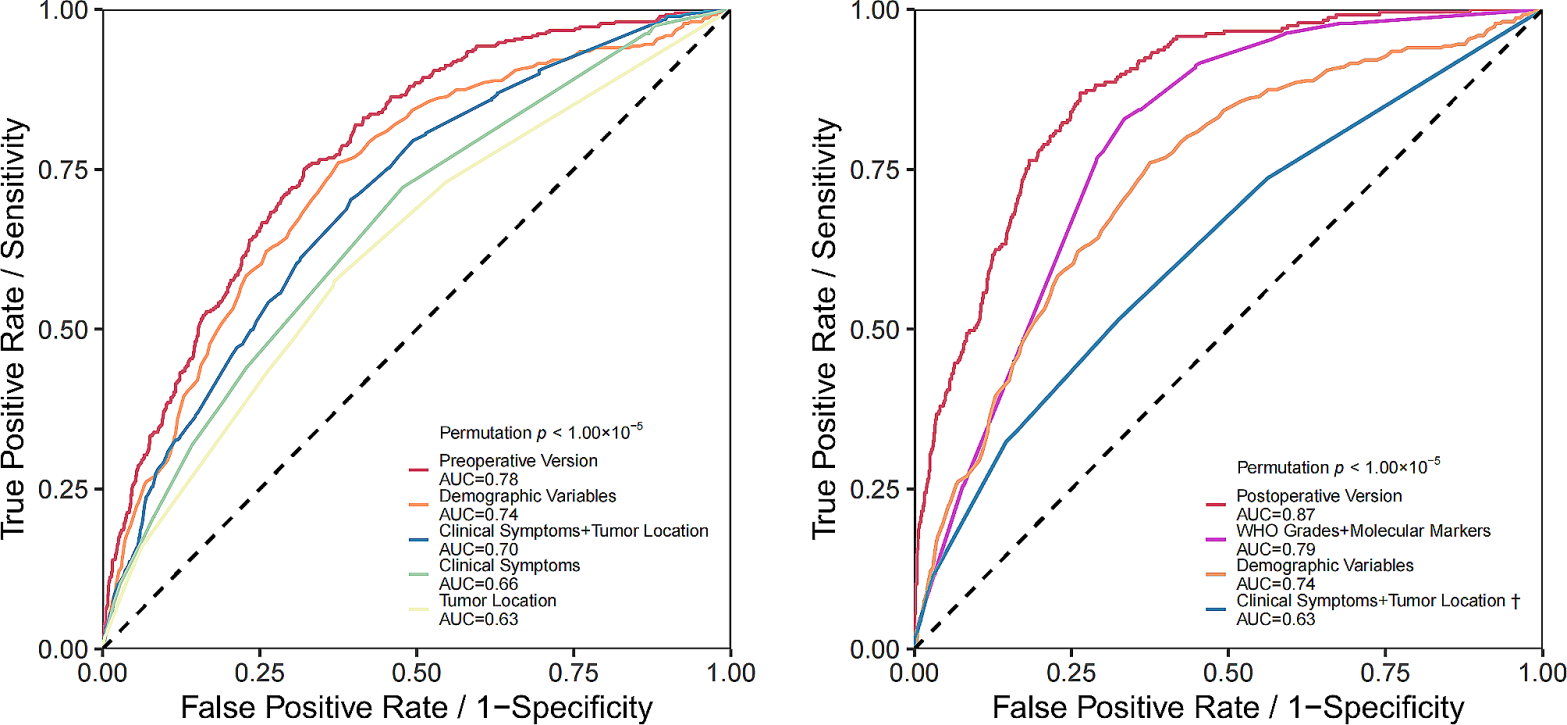
ROC curves displaying preoperative and postoperative prediction models. (**A**) ROC curve generated for the preoperative period with an AUC of 0.78. The model uses age at diagnosis, sex, and seven clinical variables including headache, vomiting, epilepsy, presence of an intracranial space-occupying lesion or tumor, muscle weakness, speech disorder, and slow reaction. Tumor location variables include the frontal lobe, occipital lobe, temporal lobe, corpus callosum, and thalamus. (**B**) ROC curve for the postoperative period showing an AUC of 0.87. The model incorporates age at diagnosis, sex, WHO grades (2 and 4), molecular parameters (GFAP+, OLIG2+, IDH-1/IDH-2+), clinical symptoms (headache, slow reaction), and tumor locations (temporal lobe, corpus callosum) † The clinical symptoms and tumor locations in panels A and B contain nonidentical variables and therefore different AUCs.

**Fig. 2:**
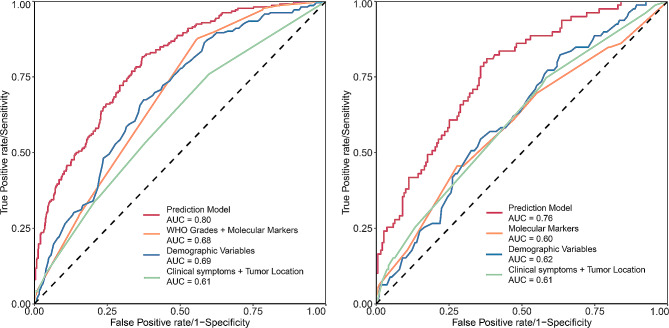
ROC curves of postoperative models derived from glioblastoma and idh wildtype subgroups. The AUCs of the postoperative prediction models developed specifically for IDH wild type (**A**) and glioblastoma (**B**) and were 0.80 and 0.76, respectively. The AUC of the postoperative models in both subgroups was significantly higher than models that included only WHO grading/molecular/clinical symptoms/tumor location information. The reason WHO grading was not included in the glioblastoma subgroup was because the glioblastomas were all grade 4

Fifteen variables were screened separately using the LASSO method to construct preoperative and postoperative models (Table 2). The AUCs of the preoperative and postoperative models were 0.78 and 0.87, respectively (Fig. 1). The nomogram (eFigure 2 in the appendix) corresponding to the postoperative model furnished convenience and maneuverability for the application of model, which was conducive to personalized medical treatment and rational therapeutic decisions. Given the absence of certain important molecular indicators, we separately tested the stability of the model within each molecular subgroup. We compared the performance of the postoperative model added with some important molecular features such as 1p/19q codeletion, telomerase reverse transcriptase (TERT), O-6-methylguanine-DNA methyltransferase (MGMT), and epidermal growth factor receptor (EGFR), respectively, with the original postoperative model by DeLong’s test and found no statistical differences (eFigure 3 in the appendix). Given the possible population heterogeneity of pediatric and older patients, we performed sensitivity analyses excluding pediatric (age less than 13 years) and older (age more than 60 years) cases, respectively. The results showed that the AUC of the preoperative and postoperative models remained at 0.78 and 0.87 after excluding some pediatric patients (eFigure 4 in the appendix). The AUCs of the preoperative and postoperative models excluding elderly patients were 0.76 and 0.88 (eFigure 5 in the appendix), respectively. The AUCs of the preoperative and postoperative models excluding pediatric and elderly patients concurrently remained 0.76 and 0.88 (eFigure 6 in the appendix), respectively, and the models had good robustness. Bootstrap-based internal validation results showed that the mean AUC values obtained from 3000 resamplings of the preoperative and postoperative models were 0.77 and 0.86, respectively (eFigure 7 in the appendix). The sensitivity analysis and internal validation yielded good results, demonstrating model robustness. Considering the poor prognosis of IDH wild-type and glioblastoma, subgroup analyses were performed specifically, and the postoperative model AUCs for the IDH wild-type and glioblastoma subgroups were 0.80 and 0.76, respectively. (Figure 2A and B). Using the developed predictive model also did a good job of identifying patients with short-term recurrence in the glioblastoma subgroup and the IDH wild-type subgroup (eFigure 8 in the appendix). The results for patients with glioblastoma are presented in eTable 1 in the appendix.

**Table 2:** Multivariable logistic regression model for predicting postoperative recurrence in glioma patients

Variables	Preoperative Model Odds Ratio (95% CI)	*p* value	Postoperative Model Odds Ratio (95% CI)	*p* value
Preoperative Period				
Age (Years at Diagnosis)	1.06 (1.05, 1.07)	< 0.0001^****^	1.04 (1.02, 1.05)	< 0.0001^****^
Sex male/female	1.37 (1.06, 1.77)	0.02^*^	1.21 (0.86, 1.71)	0.27
Headache yes/no	1.56 (1.21, 2.01)	5.90 × 10^− 4***^	1.46 (1.04, 2.04)	0.03^*^
Vomiting yes/no	1.45 (0.92, 2.29)	0.11		
Epilepsy yes/no	0.64 (0.30, 1.35)	0.24		
Intracranial Space-Occupying Lesion or Intracranial Tumor yes/no	0.37 (0.16, 0.87)	0.02^*^		
Muscle Weakness yes/no	1.53 (1.12, 2.10)	0.01^*^		
Speech Disorder yes/no	1.31 (0.89, 1.92)	0.18		
Slow Reaction yes/no	1.92 (1.03, 3.58)	0.04^*^	1.63 (0.68, 3.88)	0.27
Tumor Location (Frontal lobe) yes/no	0.82 (0.62, 1.09)	0.18		
Tumor Location (Occipital lobe) yes/no	1.14 (0.74, 1.77)	0.55		
Tumor Location (Temporal lobe) yes/no	1.40 (1.05, 1.86)	0.02^*^	1.51 (1.08, 2.12)	0.02^*^
Tumor Location (Corpus Callosum) yes/no	2.59 (1.57, 4.26)	1.85 × 10^− 4***^	2.12 (1.07, 4.20)	0.03^*^
Tumor Location (Thalamus) yes/no	5.12 (2.53, 10.40)	< 0.0001^****^		
Karnofsky Score (%)	0.99 (0.98, 1.00)	4.76 × 10^− 3**^	0.99 (0.98, 1.00)	0.06
Postoperative Period				
WHO Grade 2 yes/no			0.23 (0.10, 0.51)	3.19 × 10^− 4***^
WHO Grade 4 yes/no			2.67 (1.67, 4.26)	< 0.0001^****^
GFAP + yes/no			0.29 (0.13, 0.63)	1.91 × 10^− 3**^
OLIG2 + yes/no			0.87 (0.60, 1.27)	0.47
IDH-1/IDH-2 + yes/no			0.41 (0.24, 0.69)	7.55 × 10^− 4***^
Chemotherapy/Radiotherapy yes/no			0.24 (0.16, 0.37)	< 0.0001^****^
Karnofsky Score (%)			0.98 (0.97, 1.00)	0.08
ECOG Grade			1.09 (0.75, 1.57)	0.65

*: *p* < 0.05, **: *p* < 0.01, ***: *p* < 0.001, ****: *p* < 0.0001

### Combinations of postoperative risk factors

Based on the 15 variables in the postoperative prediction model, the statistical analyses were performed on patients who simultaneously experienced two or more risk factors. The 50 most frequent combinations of factors were identified, and the frequency distributions of these combinations were compared in patients with and without postoperative glioma recurrence. Continuous variables were dichotomized, with age at diagnosis dichotomized as < 45 years and ≥ 45 years, based on the median age at diagnosis in the study population. Thresholds for KPS scores (≤ 80 and > 80) and ECOG (< 3 and ≥ 3) were based on the level of caregiver assistance required by patients.

**Fig. 3:**
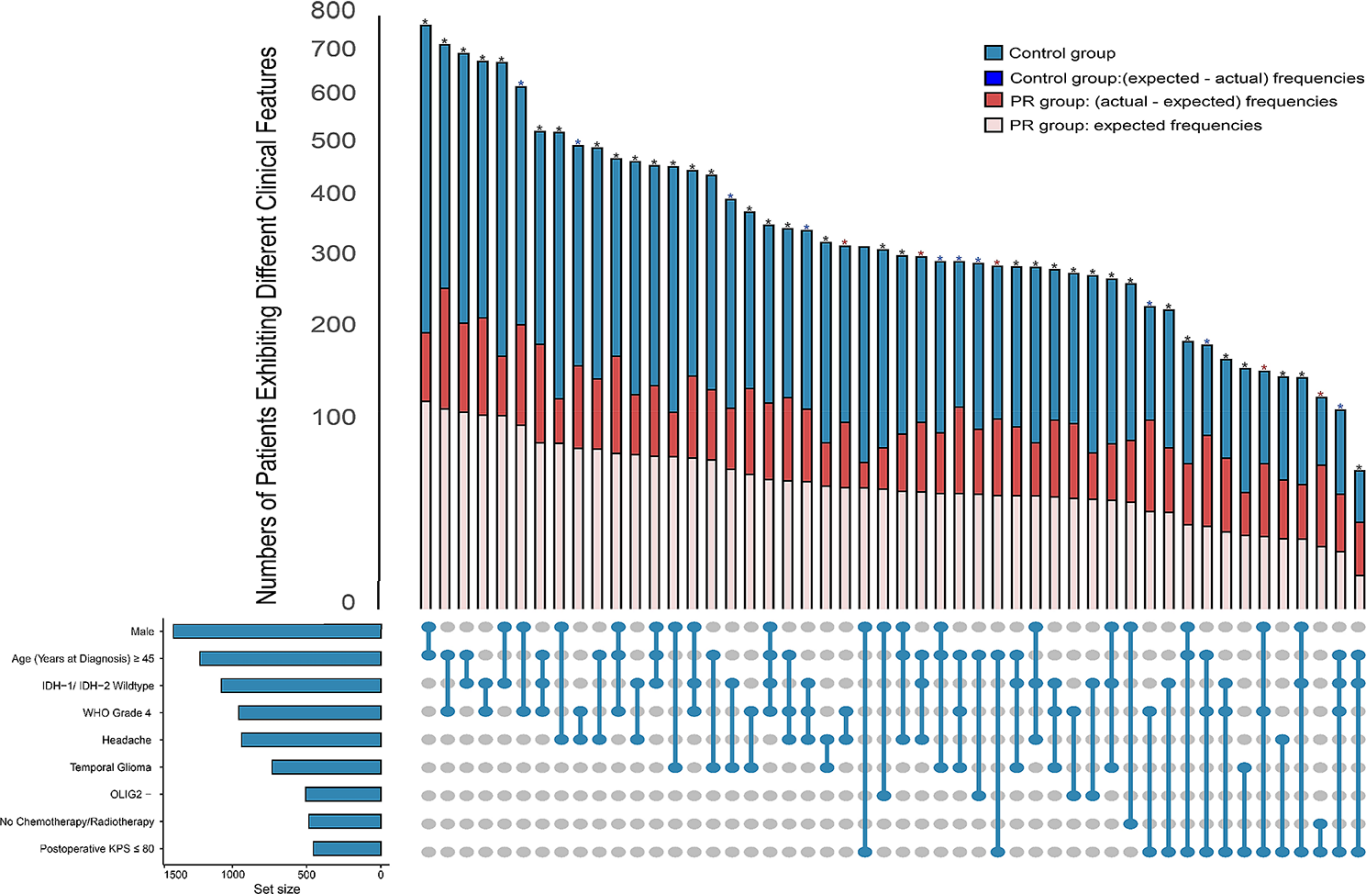
The 50 most frequent combinations of postoperative risk factors. The 50 most frequent combinations of risk factors were probed with variables derived from the postoperative version. Risk factors and their respective frequencies are shown in the lower left corner of the figure, and the dots connected by lines represent the corresponding combinations of these variables. The bar plot illustrates the frequencies of different combinations of risk factors in the postoperative recurrence (light pink and red bars) and control (bright blue and dark blue bars) groups. The light pink bars stand for the expected frequencies of postoperative recurrence calculated according to 6-month PFS rate (15.32%, 367/2396), whereas the height of the red bar indicates the numbers of the PR group in which the actual frequency was greater than the expected frequency. The red asterisks indicate combinations in the top 10% of effect sizes, whereas a blue asterisk means combinations in the top 10-30% of effect sizes with the remaining significant but not ranked in the top 30% indicated by black asterisks

A total of 14 risk factors were enrolled in the screening of characteristic combinations. To eliminate duplication of WHO grades, only WHO grade 4 was retained while WHO grade 2 was excluded, and the other four variables were not exhibited because the combinations in which they involved were not ranked among the top 50 (Fig. 3). In the figure, the connected lines of different features at the bottom represent the characteristic combinations of the 50 most common risk factors, and the corresponding bar graphs at the top show the frequencies of the different combinations of risk factors in the postoperative recurrence group (light pink and red bars) and the control group (bright blue and dark blue bars). The light pink bar indicates the expected frequency of postoperative recurrence based on the 6-month PFS rate (15.32%, 367/2396), and the height of the red bar indicates the number of people in the PR group whose actual frequency was greater than the expected frequency, and the height of the red bar responds to the magnitude of the difference in the effect size between the two groups. We compared the differences between actual and expected frequencies for 50 combinations using the chi-square test (eTable 2 in the appendix), where Benjamini-Hochberg false discovery rates (FDR) correction was used to adjust the *p*-values for multiple comparisons. Overall, the distribution of 49 combinations was statistically different in the two groups, with all adjusted *p* values < 0.05, thus potentially justifying the rationale of the variables selected for postoperative prediction. Only one combination, however, did not differ significantly, namely male plus postoperative KPS ≤ 80. Results incorporating the effect sizes calculated by Cohen’s w revealed that the top five values were different combinations of seven factors: older age, male sex, WHO grade 4, IDH wild type, No postoperative radio/chemotherapy, headache and postoperative KPS ≤ 80. The largest effect size was 0.4 for patients without radio/chemotherapy and postoperative KPS ≤ 80, followed by male patients with headaches and who are WHO Grade 4.

### Survival analysis

A total of 2,563 subjects (1,492 men [58.21%]; mean [SD] age, 44.89 [14.41] years) were enrolled in the Cox model, including 167 patients (95 men [56.89%]; mean [SD] age, 43.48 [14.12] years) who were lost to follow-up within six months. The LASSO method was used to screen and prioritize variables. Stratified Cox regression models were applied to ensure adherence to the proportional hazards (PH) assumption, with WHO grades used as important stratification variables (Table 3). The baseline cumulative hazard of the three stratified Cox regression models demonstrated an increase with higher WHO grades (eFigure 9), indicating a poorer prognosis. The comprehensive results of the three models demonstrated that age at diagnosis (level 1: hazard ratio [HR], 1.02; 95% CI, 1.00-1.04; *p* = 0.03; level 2: HR, 1.02; 95% CI, 1.01–1.03; *p* = 0.003; level 3: HR, 1.01; 95% CI, 1.01–1.02; *p* < 0.001), p53 gene mutation (level 1: HR, 2.15; 95% CI, 1.47–3.13; *p* < 0.001; level 2: HR, 1.96; 95% CI, 1.38–2.77; *p* < 0.001; level 3: HR, 1.22; 95% CI, 1.02–1.46; *p* = 0.03), IDH1/2 mutation (level 1: HR, 0.51; 95% CI, 0.34–0.76; *p* < 0.001; level 2: HR, 0.45; 95% CI, 0.32–0.64; *p* < 0.001; level 3: HR, 0.54; 95% CI, 0.39–0.74; *p* < 0.001) and postoperative KPS scores (level 1: HR, 0.98; 95% CI, 0.97–0.99; *p* < 0.001; level 2: HR, 0.98; 95% CI, 0.97–0.99; *p* < 0.001; level 3: HR, 0.99; 95% CI, 0.98–0.99; *p* < 0.001) showed consistent performance in tumors of different grades, and the differences were statistically significant. However, we also found some differences in some of the prognostic factors for tumors of different grades. Male sex (HR, 1.72; 95% CI, 1.18–2.52; *p* = 0.01) was major risk factor for postoperative glioma recurrence or death in level 1 (WHO grade 1 or 2); whereas Olig2 positivity (HR, 0.78; 95% CI, 0.64–0.95; *p* = 0.01) and postoperative chemotherapy or radiotherapy (HR, 0.38; 95% CI, 0.29–0.49; *p* < 0.001) were protective factors in level 3 (WHO grade 4). Specifically, patients with WHO grade 4 glioma had a median PFS of only 11 months. The nomograms linked to the stratified Cox regression models provided predictions of PFS probabilities at different time points (eFigure 10).

**Table 3:** Risk factors for progression free survival (PFS) in glioma patients after surgery

Variables	WHO Grade 1 and 2		WHO Grade 3		WHO Grade 4	
	Hazard Ratio (95% CI)	*p* value	Hazard Ratio (95% CI)	*p* value	Hazard Ratio (95% CI)	*p* value
Preoperative Period						
Age (Years at Diagnosis)	1.02 (1.00, 1.04)	0.03^*^	1.02 (1.01, 1.03)	3.18 × 10^− 3**^	1.01 (1.01, 1.02)	3.91 × 10^− 4***^
Sex male/female	1.72 (1.18, 2.52)	0.01^*^	0.99 (0.72, 1.36)	0.96	1.19 (0.99, 1.43)	0.06
Headache yes/no	1.09 (0.69, 1.71)	0.72	1.24 (0.89, 1.72)	0.20	1.11 (0.93, 1.32)	0.26
Epilepsy yes/no	0.95 (0.53, 1.69)	0.86	0.93 (0.49, 1.76)	0.82	0.47 (0.15, 1.50)	0.20
Intracranial Space-Occupying Lesion or Intracranial Tumor yes/no	0.81 (0.39, 1.70)	0.58	0.61 (0.24, 1.52)	0.29	1.34 (0.77, 2.32)	0.30
Illness Duration (Months)	0.99 (0.97, 1.01)	0.20	0.98 (0.96, 1.00)	0.10	1.00 (0.99, 1.01)	0.48
Tumor Location (Frontal lobe) yes/no	0.94 (0.62, 1.42)	0.77	0.84 (0.57, 1.22)	0.35	0.91 (0.74, 1.12)	0.37
Tumor Location (Occipital lobe) yes/no	1.64 (0.48, 5.55)	0.43	1.07 (0.51, 2.23)	0.86	1.18 (0.90, 1.55)	0.23
Tumor Location (Temporal lobe) yes/no	1.41 (0.91, 2.17)	0.12	1.22 (0.85, 1.74)	0.28	1.15 (0.94, 1.40)	0.17
Karnofsky Score (%)	0.98 (0.97, 1.00)	0.03^*^	1.00 (0.99, 1.01)	0.72	1.00 (0.99, 1.00)	0.48
Postoperative Period						
OLIG2 + yes/no	1.02 (0.58, 1.80)	0.94	0.79 (0.49, 1.28)	0.34	0.78 (0.64, 0.95)	0.01^*^
P53 Gene + yes/no	2.15 (1.47, 3.13)	< 0.0001****	1.96 (1.38, 2.77)	1.43 × 10^− 4***^	1.22 (1.02, 1.46)	0.03^*^
IDH1/2 + yes/no	0.51 (0.34, 0.76)	8.90 × 10^− 4***^	0.45 (0.32, 0.64)	< 0.0001^****^	0.54 (0.39, 0.74)	1.60 × 10^− 4***^
Chemotherapy/Radiotherapy yes/no	1.74 (1.00, 3.04)	0.05	0.63 (0.35, 1.13)	0.12	0.38 (0.29, 0.49)	< 0.0001****
Karnofsky Score (%)	0.98 (0.97, 0.99)	3.50 × 10^− 4***^	0.98 (0.97, 0.99)	8.75 × 10^− 4***^	0.99 (0.98, 0.99)	< 0.0001****

*: *p* < 0.05, **: *p* < 0.01, ***: *p* < 0.001, ****: *p* < 0.0001

## Discussion

In this study, logistic regression models predicting postoperative recurrence or mortality within six months and stratified Cox models for survival analysis were constructed on the basis of a comparably large sample size, with these two models exploring risk factors affecting short- and long-term PFS, respectively. In addition, both the nomograms and the combinations of risk factors grounded on postoperative logistic model had practical implications, with the nomograms providing patients with the possibility of personalized prediction schemes and optimized clinical risk management, and the risk factors combinations probing the specific effect size of different combinations of features on adverse prognosis.

The aggressive growth of gliomas often makes tumor recurrence a grim inevitability [23], putting patients at risk of recurrence or death, even following maximal safe resection. Age differed significantly across all models, with the risk of relapse or death increasing with age, as previously demonstrated [24, 25]. In addition, gliomas showed a male predominance, with males accounting for 58.21% (1492/2563) of the total study population and 64.56% (612/948) of glioblastomas. Sex-specific genetic signatures [26] have been found to predispose men to a 50% higher risk of being diagnosed with glioma than women [27]. In our study, we found that this difference was weakly correlated in WHO grade 3 tumors. Gittleman et al [28] had similar results and they found that this gender difference was not significant in patients with non-glioblastoma. We postulate that the reasons may be multifactorial, which may be related to the specific histological types of the different grades of tumors and the extent of resection. Headache, a discernible symptom potentially indicating recurrence, necessitates further clinical scrutiny. It affects 37.96% (973/2563) of the total study population, with an even higher prevalence of 50.14% (184/367) in patients experiencing postoperative recurrence. Furthermore, we have identified that preoperative intracranial space-occupying lesions serve as significant predictors of short-term recurrence in gliomas. The volume of intracranial space-occupying lesions and their location in vital functional brain regions compromises the likelihood of complete resection, a key contributor to high recurrence rate and poor prognosis [29]. Interestingly, the vast majority of intracranial space-occupying lesions are incidentally discovered during physical examination or following accidental injury, with preoperative detection of these lesions serving as a protective predictor. This underlines the proactive importance of regular physical examination in early disease detection.

The present study identified that gliomas in the temporal lobe and corpus callosum were linked to postoperative recurrence. We speculate that gliomas situated in or adjacent to the corticospinal tract, particularly high-grade gliomas, often involve the fiber tracts of the corticospinal tract, resulting in diminished muscle strength [30]. Furthermore, WHO grades and molecular markers derived from intraoperatively collected tissue sections are of great significance in delivering diagnostic and prognostic information [31]. Diffuse gliomas are invariably accompanied by relentless recurrence and inexorable progression over time [32], with WHO grade serving as an indispensable predictor, with higher grade generally presaging worse survival outcomes [33]. More than 80% of glioblastomas recur near their initial surgical site [34]. Isocitrate dehydrogenase (IDH) was used as a pivotal molecular parameter for glioma reclassification [31, 35], besides being a significant prognostic biomarker and potential therapeutic target for glioma patients [36]. Our study found high nuclear expression of Olig2 to be an important protective factor in WHO grade 4 tumors, but did not find an association with it in lower grade tumors. Olig2, a protein-coding gene commonly expressed in gliomas independent of WHO tumor grade [37]. Many previous studies have reported the important role played by Olig2 in glioblastoma cell reprogramming, genotoxic resistance, and tumor phenotypic plasticity, which may be closely related to the underlying mechanisms of tumor growth [38]. Our results are consistent with most of current studies, as a significant proportion of WHO grade 4 tumors are predominantly glioblastomas, and it is therefore important to emphasize the prognostic value of Olig2. Moreover, in our study, postoperative radiotherapy and chemotherapy was an important protective factor for WHO grade 4 tumors, but its protective effect against recurrence in low-grade tumors was not found. Indeed, we believe that this result is rational because the standard therapy for high-grade gliomas is maximal safe resection combined with adjuvant chemo/radiotherapy [4]. On the other hand, in low-grade patients, postoperative radiotherapy and chemotherapy may not be necessary in some patients with a better prognosis. For example, one study showed that [39] close monitoring may be an option for patients < 40 years of age with total tumor resection, but the decision should be made carefully after considering the patient’s condition and molecular pathology. In fact, the indications, optimal timing, and dose of postoperative radiotherapy for low-grade gliomas remain controversial [31], and treatment strategies are now usually based on the high prognostic risk of the patient, which is also consistent with our findings.

Indeed, the combinations of risk factors bring a novel perspective to the consideration of whether glioma patients harboring multiple risk factors can benefit from surgery, while also bearing the associated risks of the procedure, substantial economic costs, lengthy postoperative rehabilitation therapy, and long-term mental stress. All these factors warrant comprehensive consideration. For instance, compared to the general population, clinical decision-making in older patients often requires more complex considerations due to poorer health, multiple comorbidities, and poorer surgical tolerance [40], and we analyzed subgroups for this. The AUC of our preoperative and postoperative models reached 0.66 and 0.78 in elderly patients(see eTable 3 and eFigure 11 in the appendix), and the models may have some limitations in predicting the elderly. However, we believe this may be limited by our limited sample size. More importantly, clinical decision-making in the elderly needs to consider more complex situations as described above, not only factors related to these prognostications but also closely related to the elderly’s own physiological characteristics as well as physician’s choices. More in-depth studies of elderly glioma patients are listed in our future work. Glioma patients and their families often face significant financial burdens after diagnosis, and even among stable, high-functioning glioma survivors, financial strain and workforce morbidity are prevalent across all tumor subtypes and income levels [41]. The clinical nature of glioma, as well as other factors like treatment regimens, can directly impact brain function and may contribute to neurocognitive changes and psychological disorders [42, 43]. Therefore, clinicians should exercise caution in providing rational therapeutic recommendations to patients and their families during clinical consultations and diagnostic procedures.

### Limitations

The current study has several limitations that should be noted. Firstly, the data used in this research were collected from a single hospital, which might introduce a degree of selection bias. This could potentially affect the findings, making them less generalizable to a broader population. To address this limitation, future research should include a multicenter, prospective design with a larger cohort. Secondly, the long duration of the study coincided with updates to the WHO classification of gliomas. Due to this, histological specimens from some patients were not available, leading to the absence of some key molecular parameters in our analysis. This information could have further informed our understanding of glioma recurrence. Third, because this was a large sample cohort that lasted 8 years from 2010, many high-quality MRI images were unavailable, and at this stage, we were unable to obtain information on the extent of tumor resection or residual volume. This was known as a prognostic factor, however, the majority of clinical features critical to prognoses, such as tumor location, exhaustive molecular information, WHO classification, pre-and postoperative KPS information, and postoperative adjuvant treatment regimens, were already included in our multifactorial analysis, adjusting as much as possible for common confounders in clinical practice. We are currently working on integrating our resources and collecting as much tumor volume information as possible, and in the future, we believe this will improve the performance of the model based on existing work. Lastly, the validation of these models using independent external datasets is essential. This will ensure that the models are robust and can be reliably applied to different patient populations. In addition, there is a need for further research to evaluate the performance of these models in diverse populations and to develop more efficient deep-learning algorithms.

## Conclusions

In summary, predictive models for short-term glioma recurrence or death were developed in a substantial patient cohort, considering preoperative and postoperative perspectives. The achieved accuracies were 0.78 and 0.87, respectively, accompanied by a comprehensive evaluation of factors associated with recurrence. Key factors influencing short- and long-term progression-free survival were identified, highlighting the importance of considering individual patient circumstances in selecting appropriate treatment strategies.

### Electronic supplementary material

Below is the link to the electronic supplementary material.

Supplementary Material 1

## Data Availability

The datasets used and analysed during the current study are available from the corresponding author on reasonable request.

## Abbreviations

LASSO: Least absolute shrinkage and selection operator
PFS: Progression-free survival
CNS: Primary central nervous system
IDH: Isocitrate dehydrogenase
RANO: Response Assessment in Neuro-Oncology
AUCs: Area under the ROC curves
TERT: Telomerase reverse transcriptase
MGMT: O-6-methylguanine-DNA methyltransferase
EGFR: Epidermal growth factor receptor
PH: Proportional hazards
