# Supplementary material for: Development of preoperative and postoperative models to predict recurrence in postoperative glioma patients: a longitudinal cohort study
Source: BMC Cancer. 2024 Feb 28;24:274. doi: 10.1186/s12885-024-11996-2 (PMC10900633; doi:10.1186/s12885-024-11996-2)

Supplementary Online Content

Development of Preoperative and Postoperative Models to Predict Recurrence in Postoperative Glioma Patients: A Longitudinal Cohort Study

eTable 1. Multivariable Logistic Regression Model for Predicting Postoperative Recurrence in Patients with Glioblastoma

eTable 2. 50 risk factor combinations for comparison of actual versus expected rates

eTable 3. Multivariable Logistic Regression Model for Predicting Postoperative Recurrence in older Patients

eFigure 1. Details of Study Recruitment

eFigure 2. Nomogram of Postoperative Prediction Model

eFigure 3. ROC Curves Generated for Four Glioma Subgroups with Different Molecular Indicators

eFigure 4. ROC curves for Preoperative and Postoperative Prediction models after excluding pediatric cases

eFigure 5. ROC curves for Preoperative and Postoperative Prediction models after excluding elderly patients

eFigure 6. ROC curves for Preoperative and Postoperative Prediction models after excluding pediatric and elderly patients

eFigure 7. AUC distribution and Quantile Quantile plots for Preoperative and Postoperative models based on Bootstrap

eFigure 8. Progression-Free Survival (PFS) Curves Derived from Glioblastoma and IDH Wildtype Subgroups

eFigure 9. Baseline cumulative hazard plots for three stratified Cox regression models

eFigure 10. Nomograms of Stratified Cox Regression Models(A, B and C)

A. Nomogram of Glioma Patients with WHO Grade 1 and 2

B. Nomogram of Glioma Patients with WHO Grade 3

C. Nomogram of Glioma Patients with WHO Grade 4

eFigure 11. ROC curves for Preoperative and Postoperative Prediction models for subgroups of Elderly gliomas

eTable 1 Multivariable Logistic Regression Model for Predicting Postoperative Recurrence in Patients with Glioblastoma

Variables	Odds Ratio (95% CI)	*p* value
**Preoperative Period**		
**Age (Years at Diagnosis)**	1.04 (1.01, 1.07)	0.02*
**Male yes/no**	1.88 (0.91, 3.91)	0.09
**Headache yes/no**	3.59 (1.60, 8.06)	1.93×10^{-3}**
**Impaired Consciousness yes/no**	4.62 (1.47, 14.52)	0.01*
**Slow Reaction yes/no**	4.82 (0.68, 34.30)	0.12
**Visual Impairment yes/no**	0.05 (2.90×10^{-3}, 1.01)	0.05
**Tumor Location (Insular lobe) yes/no**	3.44 (0.81, 14.52)	0.09
**Tumor Location (Ventricle) yes/no**	6.30 (0.45, 88.40)	0.17
**Postoperative Period**		
**GFAP+ yes/no**	0.27 (0.04, 2.00)	0.20
**OLIG2+ yes/no**	0.66 (0.31, 1.42)	0.29
**Ki-67 ≥ 20% yes/no**	0.57 (0.27, 1,18)	0.13
**1p/19q Codeletion yes/no**	4.26 (0.50, 36.43)	0.19
**MGMT+ yes/no**	0.93 (0.46, 1.91)	0.85
**Chemotherapy/ Radiotherapy yes/no**	0.16 (0.05, 0.46)	7.59×10^{-4}***
**Karnofsky Score (%)**	0.97 (0.94, 0.99)	1.50×10^{-3}**

*: $p < 0.05$, **: $p < 0.01$, ***: $p < 0.001$, ****: $p < 0.0001$.

eTable 2 50 risk factor combinations for comparison of actual versus expected rates

Serial No.	Male	Age at Diagnosis > 45	Headache	Temporal Glioma	IDH-1/IDH-2 wildtype	WHO Grade 4	OLIG2 -	Chemotherapy/ Radiotherapy -	Postoperative KPS ⩽ 80	χ^2	P_{FDR} value*	Cohen's w
1	√	√								22.84	<0.001	0.12
2		√				√				74.89	<0.001	0.23
3		√			√					39.91	<0.001	0.17
4					√	√				48.41	<0.001	0.19
5	√				√					17.39	<0.001	0.11
6	√					√				57.54	<0.001	0.25
7		√			√	√				47.57	<0.001	0.20
8	√		√							11.4	0.001	0.14
9			√			√				42.05	<0.001	0.27
10		√	√							24.59	<0.001	0.16
11	√	√				√				45.33	<0.001	0.20
12			√		√					17.53	<0.001	0.14
13	√	√			√					24.21	<0.001	0.16
14	√			√						9.95	0.002	0.11
15	√				√	√				33.36	<0.001	0.19
16		√		√						24.36	<0.001	0.16
17				√	√					25.77	<0.001	0.26
18				√		√				34.66	<0.001	0.19
19	√	√			√	√				28.64	<0.001	0.19
20		√	√			√				33.64	<0.001	0.20
21			√		√	√				30.69	<0.001	0.26
22			√	√						9.06	0.003	0.11
23	√		√			√				38.59	<0.001	0.39
24	√								√	3.14	0.077	0.07
25	√						√			8.54	0.003	0.12
26	√	√	√							16.11	<0.001	0.16
27		√	√		√					29.26	<0.001	0.28
28	√	√		√						21.63	<0.001	0.23
29		√		√		√				37.48	<0.001	0.23
30		√					√			26.09	<0.001	0.27
31		√							√	34.89	<0.001	0.29
32		√		√	√					23.10	<0.001	0.19
33	√		√		√					17.38	<0.001	0.23
34				√	√	√				28.48	<0.001	0.21
35						√	√			26.76	<0.001	0.20
36					√		√			10.73	0.001	0.14
37	√			√	√					16.07	<0.001	0.17
38	√							√		19.53	<0.001	0.19
39						√			√	40.78	<0.001	0.26
40					√				√	20.45	<0.001	0.20

41	√	√						√	17.22	<0.001	0.18
42		√				√		√	38.36	<0.001	0.26
43					√	√		√	24.49	<0.001	0.22
44				√				√	8.41	0.004	0.14
45	√					√		√	29.27	<0.001	0.34
46			√					√	15.07	<0.001	0.17
47	√				√			√	13.95	<0.001	0.17
48							√	√	37.74	<0.001	0.40
49		√			√	√		√	15.73	<0.001	0.25
50		√			√			√	11.61	0.001	0.17

eTable 3 Multivariable Logistic Regression Model for Predicting Postoperative Recurrence in older Patients

Variables	Preoperative Model Odds Ratio (95% CI)	p value	Postoperative Model Odds Ratio (95% CI)	p value
**Preoperative Period**				
**Age (Years at Diagnosis)**	1.06 (1.01, 1.12)	**0.01***	1.05 (0.98, 1.12)	0.14
**Sex male/female**	1.02 (0.65, 1.57)	0.94	1.13 (0.63, 2.01)	0.69
**Headache yes/no**	1.16 (0.74, 1.82)	0.51	1.23 (0.70, 2.16)	0.47
**Vomiting yes/no**	1.58 (0.57, 4.23)	0.36		
**Intracranial Space-Occupying Lesion or Intracranial Tumor yes/no**	0.43 (0.07, 1.70)	0.29		
**Muscle Weakness yes/no**	1.84 (1.15, 2.96)	0.01*		
**Speech Disorder yes/no**	1.35 (0.73, 2.45)	0.33		
**Slow Reaction yes/no**	1.83 (0.84, 4.01)	0.13	1.53 (0.51, 4.55)	0.44
**Tumor Location (Frontal lobe) yes/no**	1.02 (0.84, 4.01)	0.93		
**Tumor Location (Occipital lobe) yes/no**	1.08 (0.55, 2.10)	0.82		
**Tumor Location (Temporal lobe) yes/no**	1.22 (0.74, 2.00)	0.43	1.47 (0.84, 2.60)	0.18
**Tumor Location (Corpus Callosum) yes/no**	2.96 (1.21, 7.53)	0.02*	3.45 (1.05, 11.62)	0.04*
**Tumor Location (Thalamus) yes/no**	4.62 (0.41, 1.04)	0.23		
**Karnofsky Score (%)**	0.99 (0.98, 1.00)	0.17	0.99 (0.97, 1.01)	0.41
**Postoperative Period**				
**WHO Grade 2 yes/no**			0.21 (0.00, 32.7)	0.98
**WHO Grade 4 yes/no**			1.55 (0.71, 3.58)	0.29
**OLIG2+ yes/no**			0.66 (0.34, 1.26)	0.20
**IDH-1/IDH-2 + yes/no**			0.80 (0.29, 2.10)	0.66
**Chemotherapy/ Radiotherapy yes/no**			0.19 (0.09, 0.38)	< 0.0001****
**Karnofsky Score (%)**			0.99 (0.96, 1.02)	0.35
**ECOG Grade**			0.84 (0.45, 1.51)	0.56

***: $p < 0.05$, **: $p < 0.01$, ***: $p < 0.001$, ****: $p < 0.0001$.**

eFigure 1. Details of Study Recruitment

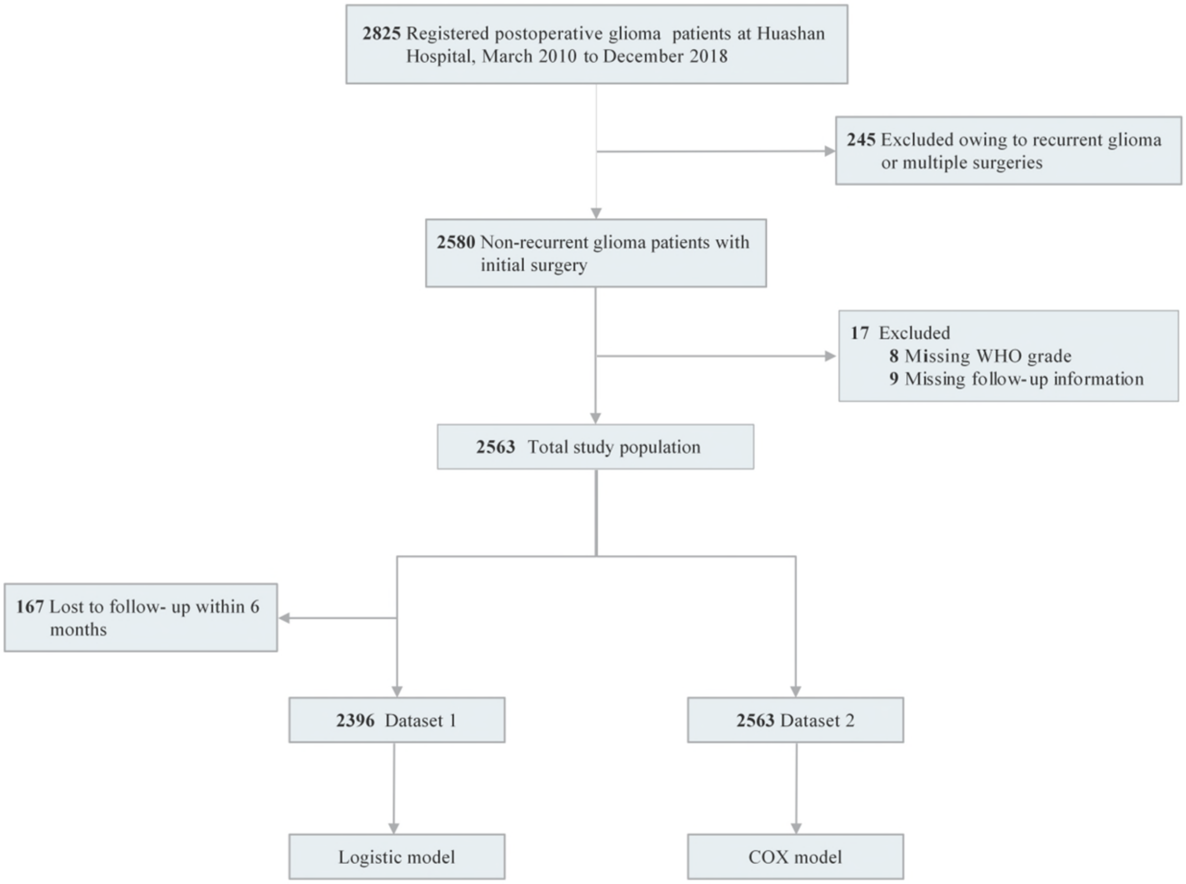

eFigure 2. Nomogram of Postoperative Prediction Model

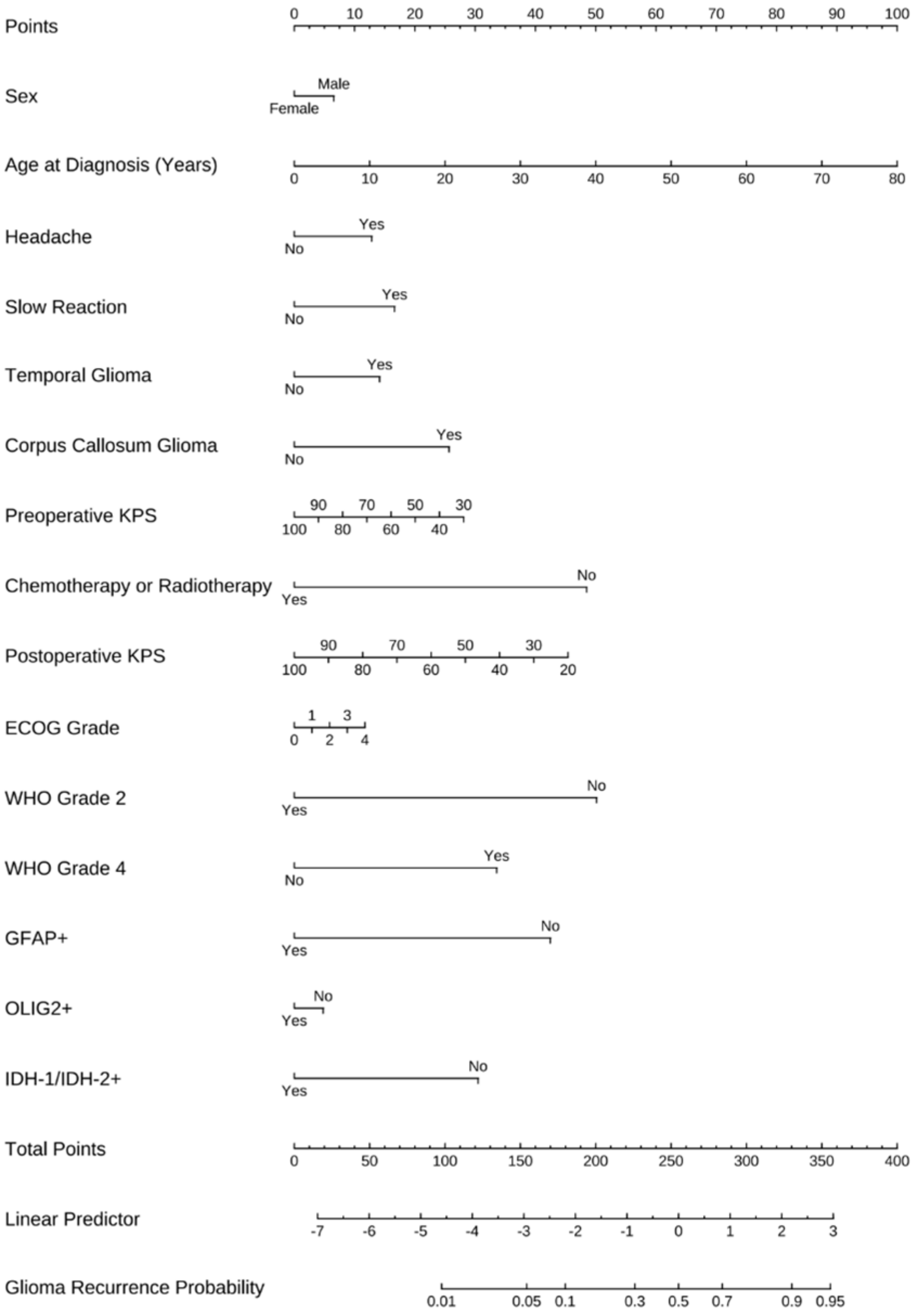

eFigure 3. ROC Curves Generated for Four Glioma Subgroups with Different Molecular Indicators

The postoperative models in subplots A, B, C and D are based on four different population subgroups with the following numbers: 1037, 919, 1793 and 145, but the 15variables included are the same as the postoperative model in Table 2.

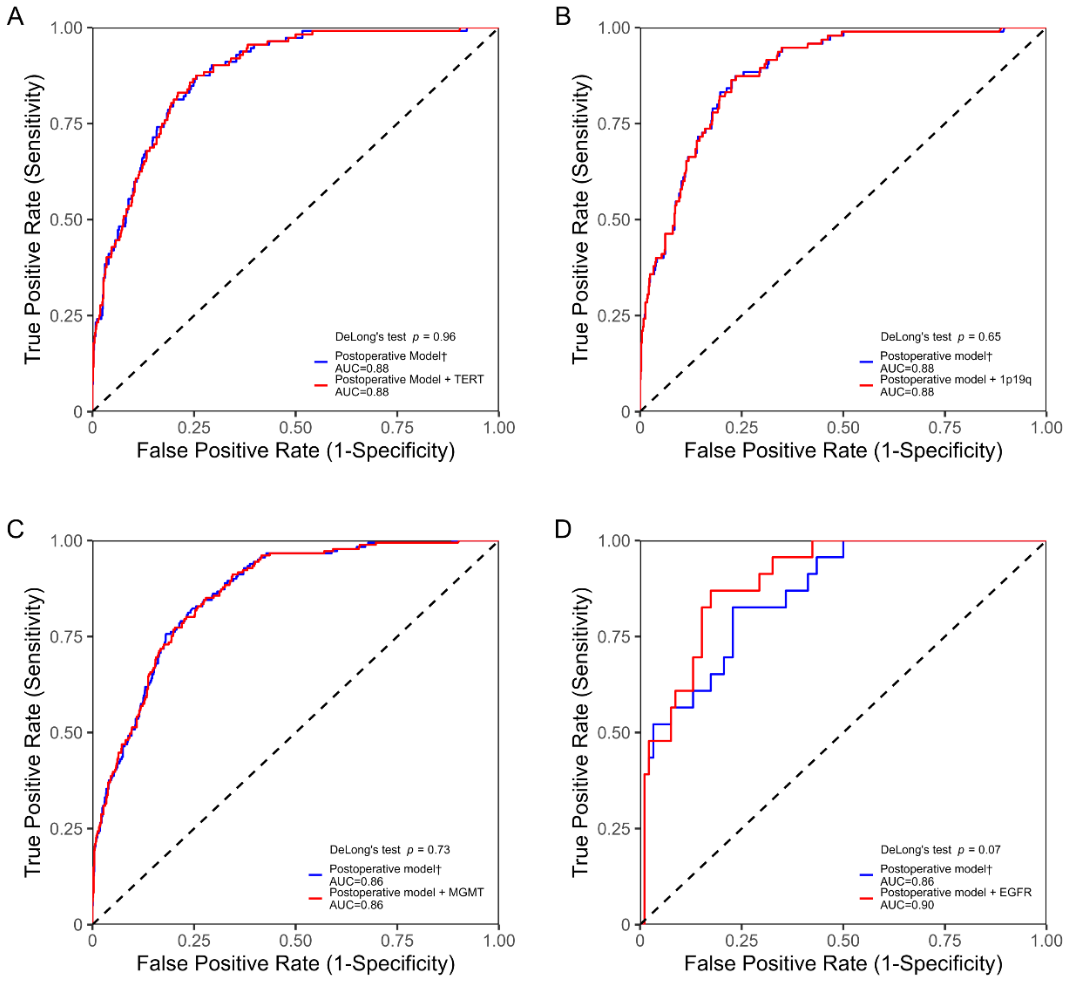

eFigure 4. ROC curves for Preoperative and Postoperative Prediction models after excluding pediatric cases

Receiver operating characteristic (ROC) curves were generated for Preoperative (A) and Postoperative (B) model based on multiple logistic regression. The AUC for Preoperative (A) and Postoperative (B) model were 0.78 and 0.87 respectively.

A

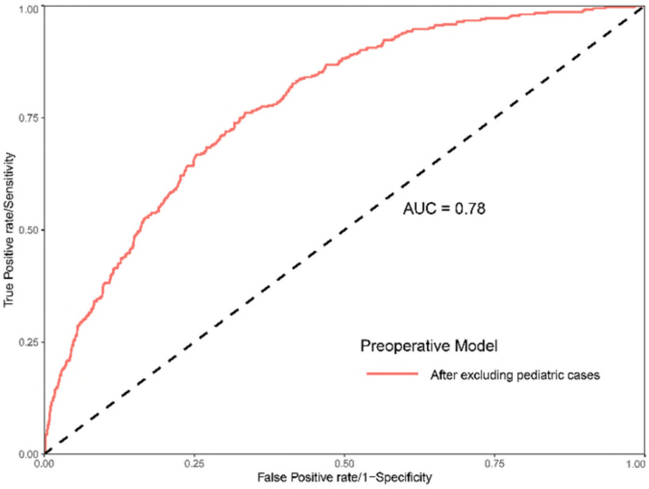

B

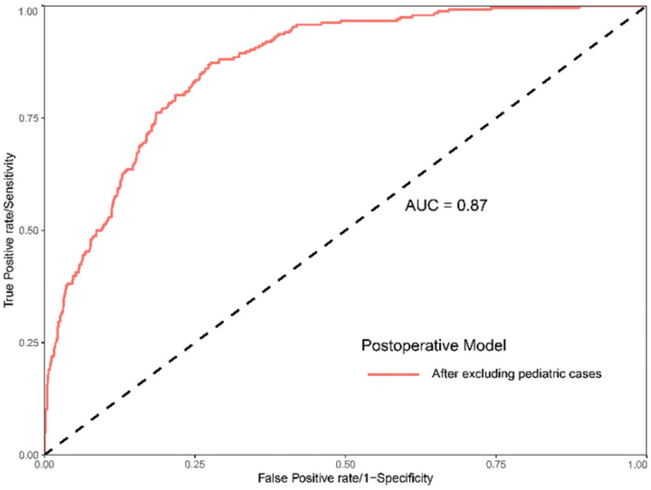

eFigure 5. ROC curves for Preoperative and Postoperative Prediction models after excluding elderly patients

Receiver operating characteristic (ROC) curves were generated for Preoperative (A) and Postoperative (B) model based on multiple logistic regression. The AUC for Preoperative (A) and Postoperative (B) model were 0.76 and 0.88 respectively.

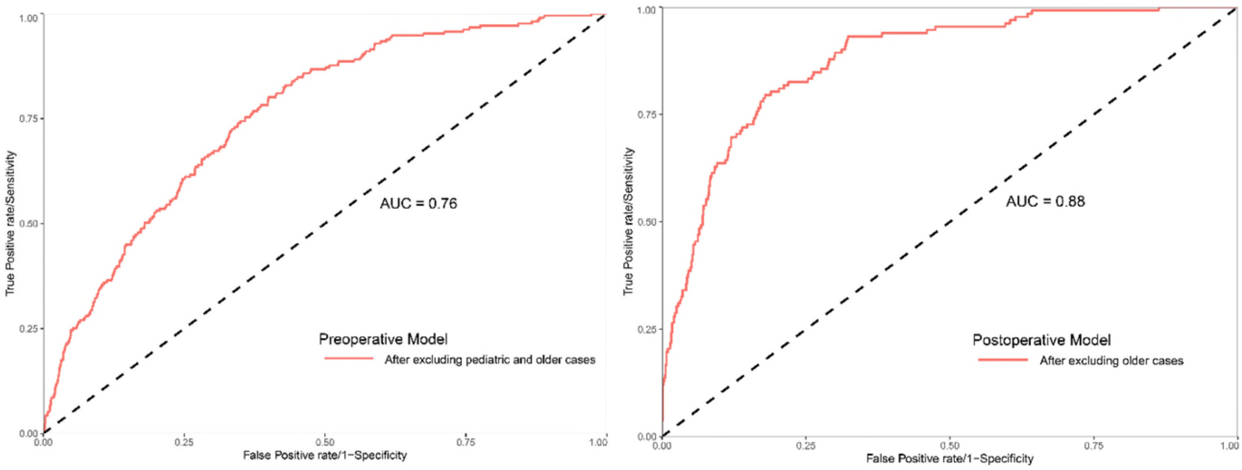

eFigure 6. ROC curves for Preoperative and Postoperative Prediction models after excluding pediatric and elderly patients

Receiver operating characteristic (ROC) curves were generated for Preoperative (A) and Postoperative (B) model based on multiple logistic regression. The AUC for Preoperative (A) and Postoperative (B) model were 0.76 and 0.88 respectively.

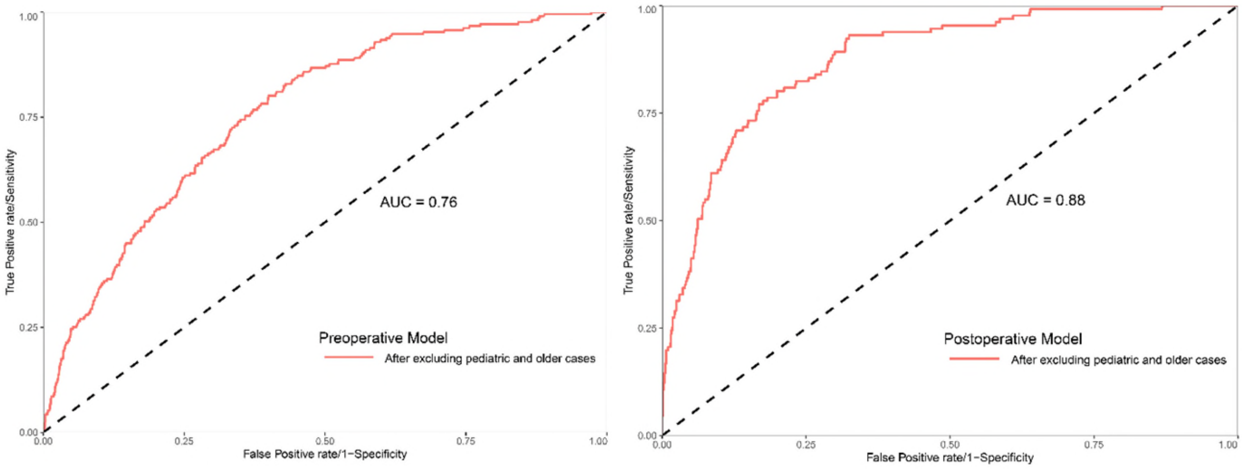

eFigure 7. AUC distribution and Quantile Quantile plots for Preoperative and Postoperative models based on Bootstrap

The AUC distribution and Quantile Quantile plots were generated for the preoperative (A) and postoperative (B) models based on internal validation with 3000 resamples. The mean AUC for multiple resampling was 0.77 and 0.86 for the preoperative (A) and postoperative (B) models, respectively.

A

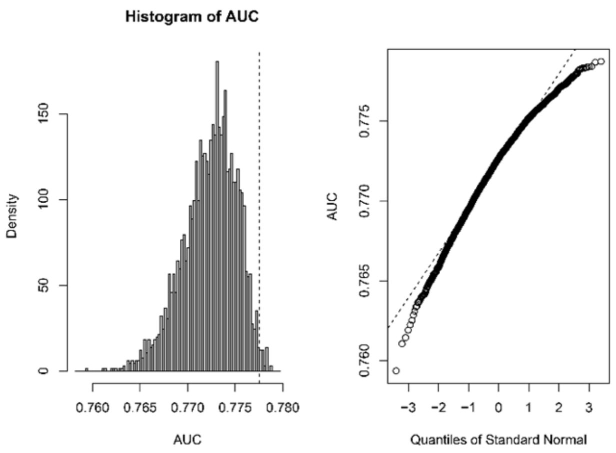

B

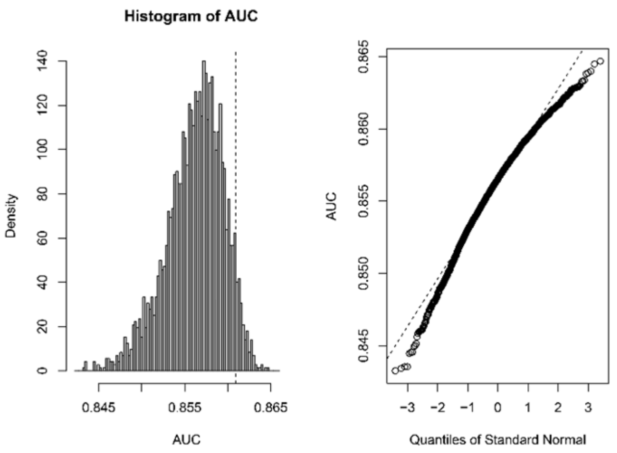

eFigure 8. Progression-Free Survival (PFS) Curves Derived from Glioblastoma and IDH Wildtype Subgroups

Progression-free survival plots were generated for the glioblastoma (A) and IDH wild-type (B) subgroups, respectively, with the postoperative recurrence group in red and the control group in blue based on postoperative predictive model classification.

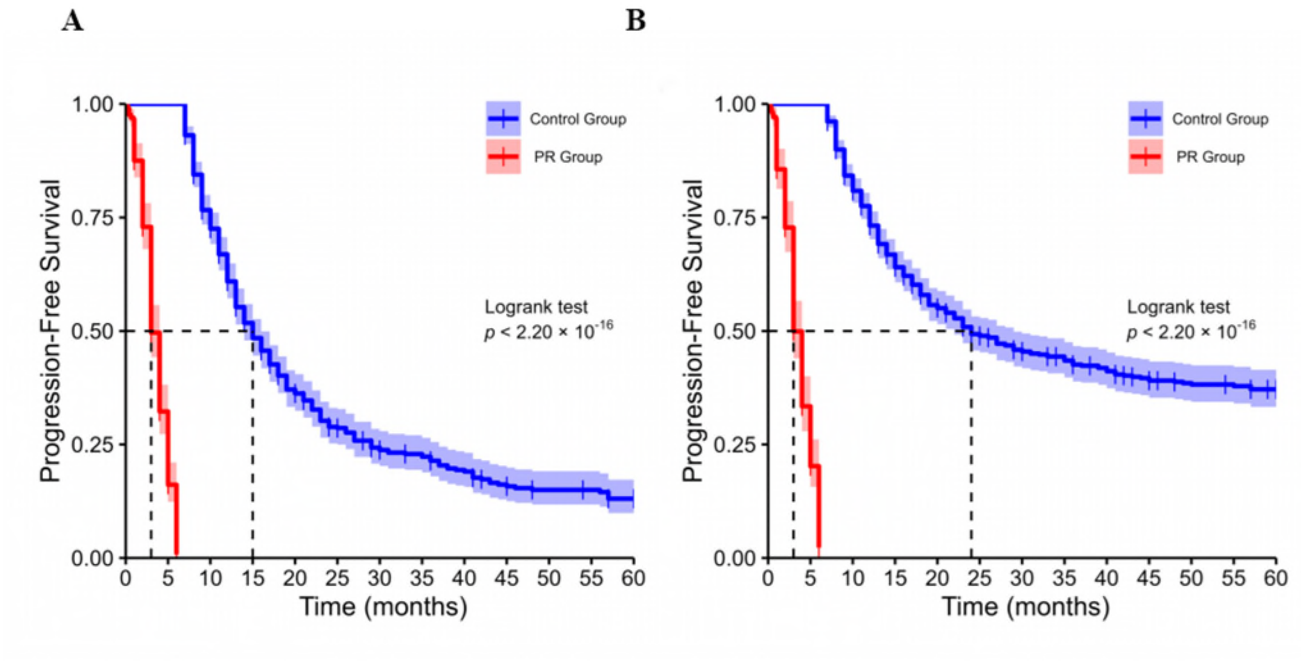

eFigure 9. Baseline cumulative hazard plots for three stratified Cox regression models

Baseline cumulative hazard plots were prepared for the three stratified COX regression models (green line, WHO grades 1 and 2; blue line, WHO grade 3; red line, WHO grade 4) relevant to progression-free survival, where baseline cumulative hazard increased with increasing WHO grade.

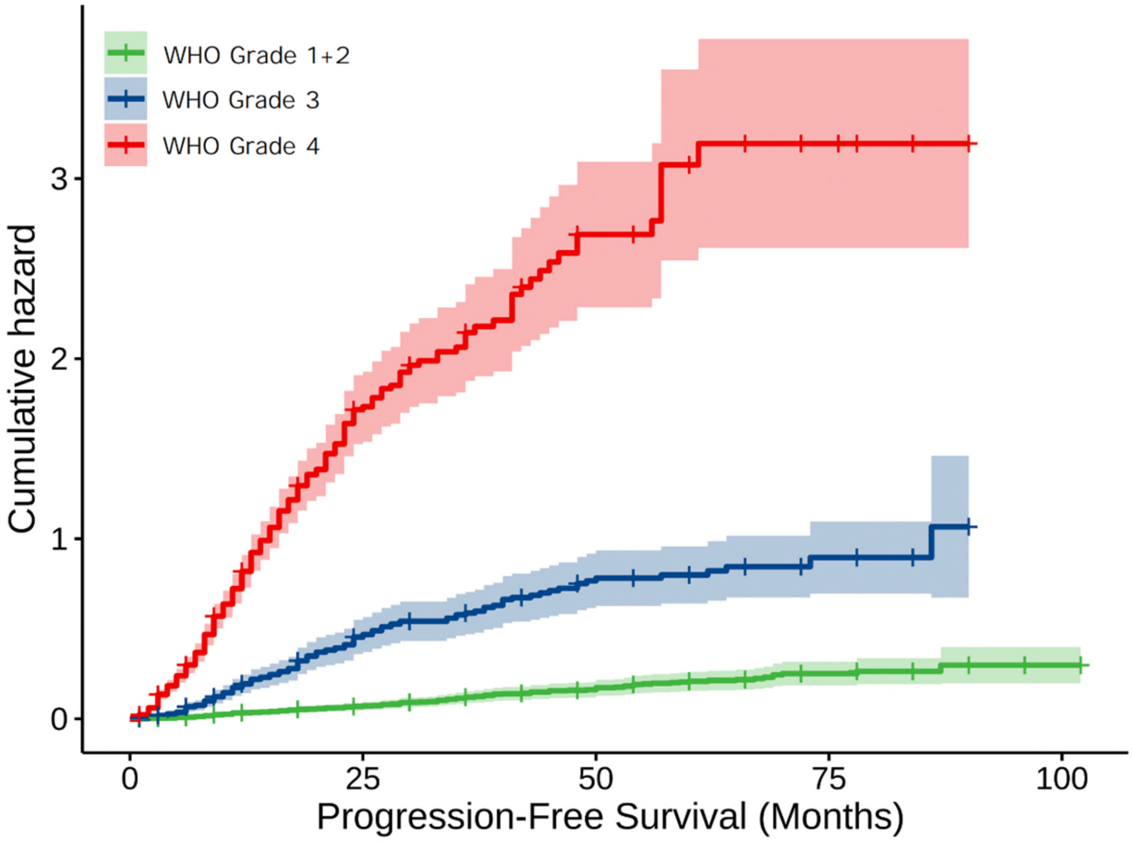

eFigure 10. Nomograms of Stratified Cox Regression Models(A, B and C)

A. Nomogram of Glioma Patients with WHO Grade 1 and 2

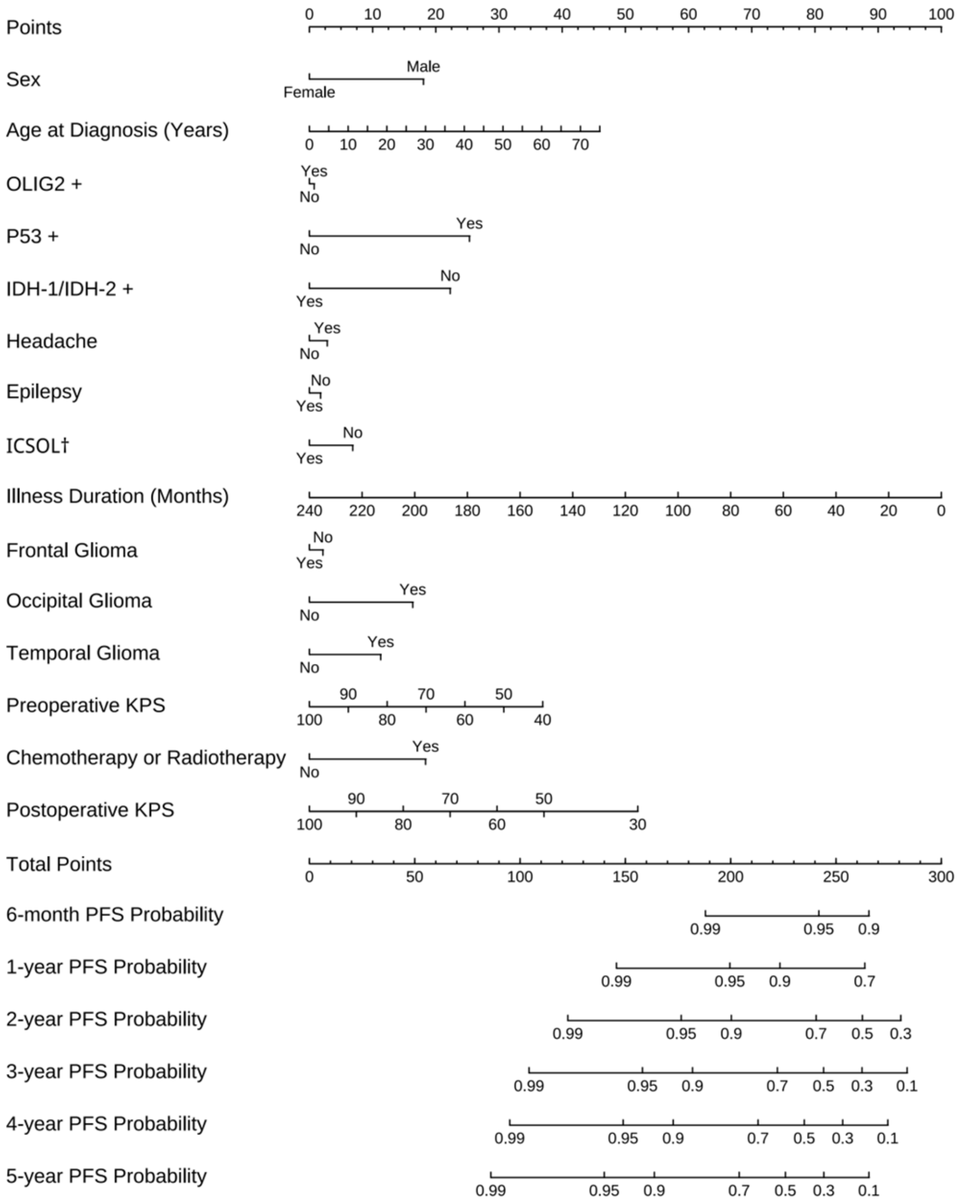

† ICSOL: intracranial space-occupying lesion or intracranial tumor

B. Nomogram of Glioma Patients with WHO Grade 3

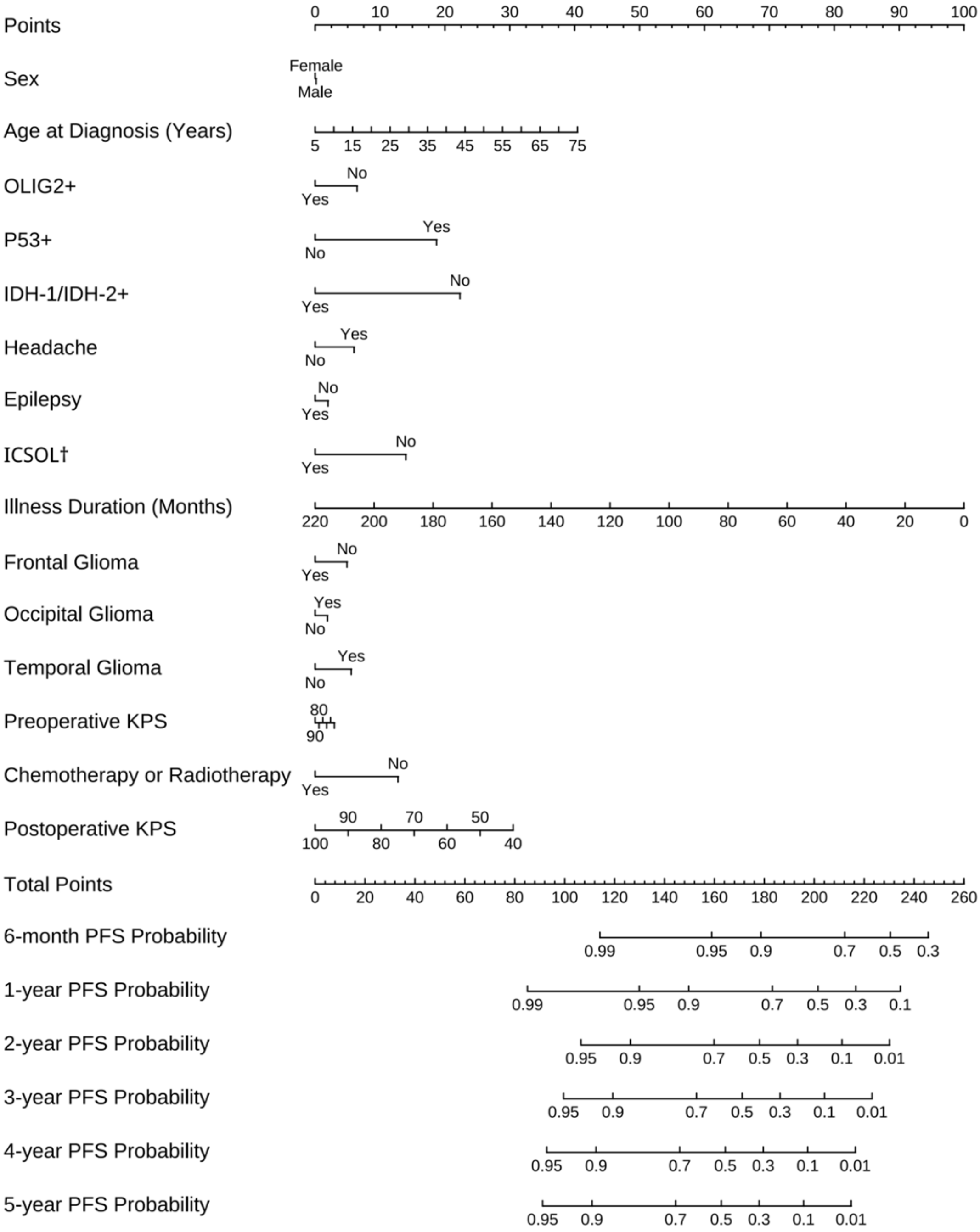

† ICSOL: intracranial space-occupying lesion or intracranial tumor

C. Nomogram of Glioma Patients with WHO Grade 4

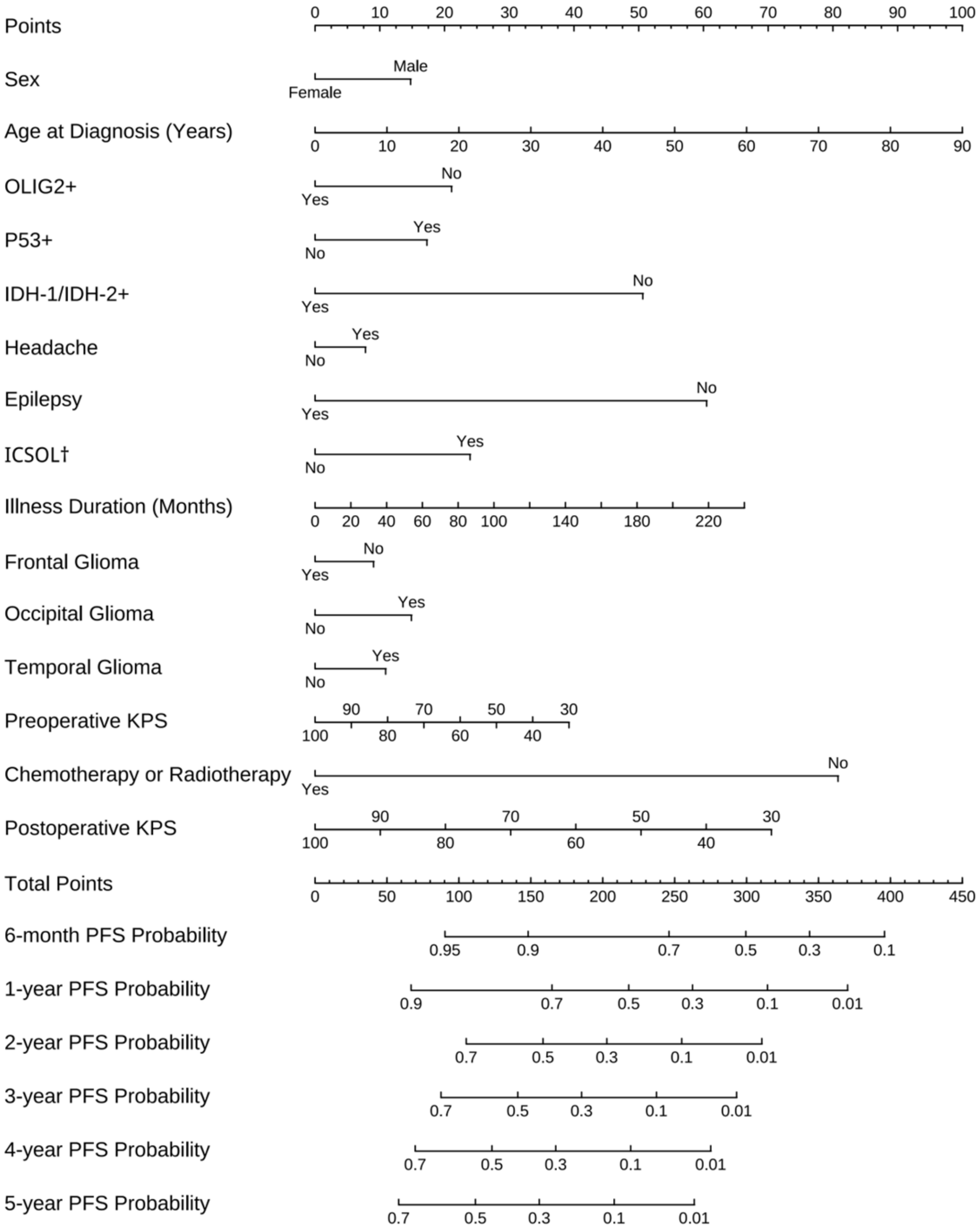

† ICSOL: intracranial space-occupying lesion or intracranial tumor

eFigure 11. ROC curves for Preoperative and Postoperative Prediction models for subgroups of Elderly gliomas

Receiver operating characteristic (ROC) curves were generated for Preoperative (A) and Postoperative (B) model based on multiple logistic regression. The AUC for Preoperative (A) and Postoperative (B) model were 0.66 and 0.78 respectively.

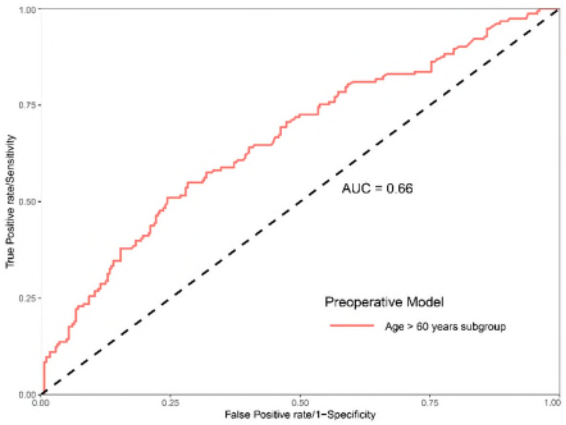

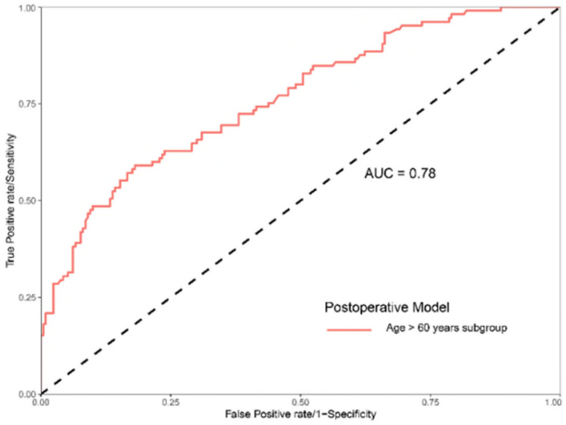

Supplement: Supplementary file 1 — Supplementary Material 1 [file 12885_2024_11996_MOESM1_ESM.pdf]
